# Screen Time and Autism Spectrum Disorder: A Systematic Review and Meta-Analysis

**DOI:** 10.1001/jamanetworkopen.2023.46775

**Published:** 2023-12-08

**Authors:** Yaakov Ophir, Hananel Rosenberg, Refael Tikochinski, Shani Dalyot, Yuliya Lipshits-Braziler

**Affiliations:** 1 Department of Education, Ariel University, Ariel, Israel; 2 Centre for Human Inspired Artificial Intelligence, University of Cambridge, Cambridge, United Kingdom; 3 School of Communication, Ariel University, Ariel, Israel; 4 Faculty of Data and Decision Sciences, Technion–Israel Institute of Technology, Haifa, Israel; 5 Communications Department, Sapir Academic College, Hof Ashkelon, Israel; 6 Seymour Fox School of Education, Hebrew University of Jerusalem, Jerusalem, Israel

## Abstract

**Importance:**

Contemporary studies raise concerns regarding the implications of excessive screen time on the development of autism spectrum disorder (ASD). However, the existing literature consists of mixed and unquantified findings.

**Objective:**

To conduct a systematic review and meta-analyis of the association between screen time and ASD.

**Data Sources:**

A search was conducted in the PubMed, PsycNET, and ProQuest Dissertation & Theses Global databases for studies published up to May 1, 2023.

**Study Selection:**

The search was conducted independently by 2 authors. Included studies comprised empirical, peer-reviewed articles or dissertations published in English with statistics from which relevant effect sizes could be calculated. Discrepancies were resolved by consensus.

**Data Extraction and Synthesis:**

This study followed the Preferred Reporting Items for Systematic Reviews and Meta-Analyses (PRISMA) reporting guideline. Two authors independently coded all titles and abstracts, reviewed full-text articles against the inclusion and exclusion criteria, and resolved all discrepancies by consensus. Effect sizes were transformed into log odds ratios (ORs) and analyzed using a random-effects meta-analysis and mixed-effects meta-regression. Study quality was assessed using the Grading of Recommendations, Assessment, Development, and Evaluations (GRADE) approach. Publication bias was tested via the Egger *z* test for funnel plot asymmetry. Data analysis was performed in June 2023.

**Main Outcomes and Measures:**

The 2 main variables of interest in this study were screen time and ASD. Screen time was defined as hours of screen use per day or per week, and ASD was defined as an ASD clinical diagnosis (yes or no) or ASD symptoms. The meta-regression considered screen type (ie, general use of screens, television, video games, computers, smartphones, and social media), age group (children vs adults or heterogenous age groups), and type of ASD measure (clinical diagnosis vs ASD symptoms).

**Results:**

Of the 4682 records identified, 46 studies with a total of 562 131 participants met the inclusion criteria. The studies were observational (5 were longitudinal and 41 were cross-sectional) and included 66 relevant effect sizes. The meta-analysis resulted in a positive summary effect size (log OR, 0.54 [95% CI, 0.34 to 0.74]). A trim-and-fill correction for a significant publication bias (Egger *z* = 2.15; *P* = .03) resulted in a substantially decreased and nonsignificant effect size (log OR, 0.22 [95% CI, −0.004 to 0.44]). The meta-regression results suggested that the positive summary effect size was only significant in studies targeting general screen use (β [SE] = 0.73 [0.34]; *t*_58_ = 2.10; *P* = .03). This effect size was most dominant in studies of children (log OR, 0.98 [95% CI, 0.66 to 1.29]). Interestingly, a negative summary effect size was observed in studies investigating associations between social media and ASD (log OR, −1.24 [95% CI, −1.51 to −0.96]).

**Conclusions and Relevance:**

The findings of this systematic review and meta-analysis suggest that the proclaimed association between screen use and ASD is not sufficiently supported in the existing literature. Although excessive screen use may pose developmental risks, the mixed findings, the small effect sizes (especially when considering the observed publication bias), and the correlational nature of the available research require further scientific investigation. These findings also do not rule out the complementary hypothesis that children with ASD may prioritize screen activities to avoid social challenges.

## Key Points

**Question:**

Is there an association between screen time and autism spectrum disorder (ASD)?

**Findings:**

In this systematic review and meta-analysis of 46 of 4682 observational studies, a statistically significant association was found between screen time and ASD, in particular among studies that examined general screen use among children. However, when accounting for publication bias, the findings were no longer statistically significant.

**Meaning:**

These findings suggest that excessive screen time may be associated with negative developmental outcomes; however, the observational nature and publication bias of the included studies render these findings inconclusive.

## Teaser

This systematic review and meta-analysis examines the association between screen time and autism spectrum disorder.

## Introduction

The ever-increasing rates of autism spectrum disorder (ASD),^1,2^ a neurodevelopmental condition characterized by difficulties in interpersonal interactions and communication, as well as restricted and repetitive behaviors, are a major concern in pediatrics. Several explanations have been proposed for this increased prevalence,^3,4^ including the global emergence of screen-based devices (eg, smartphones, tablets) and their ubiquitous use among young children, including infants.^5^ Corresponding to a longstanding concern in media psychology termed the *displacement hypothesis*,^6^ contemporary scholars warn that excessive screen use may come at the expense of positive and vital real-life experiences, such as interpersonal interactions, outdoor and sporting events, and educational activities.^7,8^ According to this hypothesis, screen use contributes to young children being less active, less verbal, and less social than children of previous generations, essentially increasing their risk of experiencing developmental delays, behavioral problems, and ASD symptoms.^9,10,11,12,13^

Although this concern, along with multiple other screen-related risks,^14^ warrants the periodic formulation of screen use guidelines for parents (such as the recent recommendations issued by the World Health Organization^15^), its empirical foundations remain unclear. To date, very few longitudinal studies have been conducted on this topic, and the picture arising from the existing, mostly cross-sectional literature is ambiguous and requires further examination.^16,17,18^

Before undertaking the current study, we identified 2 systematic reviews that addressed the association between screen time and ASD.^17,18^ Indeed, these reviews focused on the opposite direction of this association—that is, they explored the hypothesis that children with ASD would be more attracted than their peers to screen activities because these activities allow them to avoid real-life communication challenges. However, the results of these reviews also may be relevant to our research question, because they relied mostly on bidirectional correlational studies.

A 2018 systematic review by Stiller and Mößle^17^ that included 47 studies was inconclusive. Some studies indicated that children with ASD have increased screen time, whereas other studies suggested that children without ASD have increased screen time.^17^ The 2019 systematic review by Slobodin et al^18^ implemented a more rigid inclusion criterion and included only studies that compared participants with diagnosed ASD with nondiagnosed participants. That review yielded 16 relevant studies, of which 14 pointed to a consistent trend whereby children with ASD indeed had increased screen time.^18^ Nevertheless, Slobodin et al emphasized that the wide variability in the populations and methodologies in the included studies limited their finding, and they stated that “there are no data to confirm or refute a causal relationship between ASD and screen use.”^18^^(p309)^

Apart from these limitations and the mixed findings of Stiller and Mößle,^17^ we identified 3 additional gaps in the literature. First, the latest search of the literature ended in April 2018, while screen use has only become more popular over the years, especially during the COVID-19 pandemic that shifted peoples’ activity to screen-based platforms.^19^ Second, none of the available reviews included a quantitative evaluation of the association between screen time and ASD using a meta-analysis procedure. Finally, although several moderating factors were suggested in these reviews (eg, to explain mixed findings), none included a designated analysis that could shed light on the moderating role of these factors in the association between screen time and ASD.

Considering these gaps, we performed an updated systematic review and, to our knowledge, the first meta-analysis of the literature accumulated on the bidirectional association between screen time and ASD. In addition, this study also implemented meta-regression analyses to explore potential moderating factors that may be involved in this association. Specifically, the following 3 salient variables that distinguished the collected studies from one another were examined: (1) the type of screen device or screen activity (eg, smartphones, social media), (2) the age of screen users, and (3) the type of ASD measure, whether it reflected an ASD clinical diagnosis or symptoms or behaviors typical to ASD. Although these procedures cannot compensate for the absence of experimental studies, their results may shed light on this concerning association between screen time and ASD.

## Methods

This systematic review and meta-analysis followed the Preferred Reporting Items for Systematic Reviews and Meta-Analyses (PRISMA) guideline. The 2 main variables of interest were screen time (ie, hours of screen use per day or per week) and ASD. The ASD variable consisted of 2 measures: (1) a binary variable (yes or no) that indicated the presence of a clinical diagnosis of ASD and (2) a continuous variable that indicated the existence of symptoms or behaviors typical to ASD (but may not necessarily indicate the existence of a clinical diagnosis).

### Search Strategy

A systematic search of relevant studies published up to May 1, 2023, was conducted in the 2 prominent databases in medicine and psychology: PubMed and PsycNET. A complementary search for unpublished work was conducted in the ProQuest Dissertation & Theses Global database. The search did not contain restrictions on publication type or language. Terms related to screen time and ASD were searched in all available database search fields, except in PubMed, which allowed a narrowed search within the study title and abstract (assuming that relevant articles mentioned the search terms in these sections).

For ASD, the search terms used were *autism* and *ASD*. For screen time, the search terms used were *computer*, *media*, *mobile media*, *mobile phone*, *phone*, *screen time*, *smartphone*, *social media*, *television*, and *video games*. This list was discussed among and consolidated by all authors, and it includes and extends the list used in the most recent systematic review on this topic.^18^ During the search, each term for screen time was coupled with the terms for ASD; that is, the screen time terms were searched twice, once with *ASD* and once with *autism spectrum disorder*. Two authors (R.T. and S.D.) independently coded all titles and abstracts, reviewed full-text articles against the inclusion and exclusion criteria, and resolved all discrepancies by consensus.

After duplicate records were removed, the PubMed and PsycNET searches yielded 4677 articles. These articles examined the association between ASD and the following: computers (2884 articles), media (922 articles), mobile media (7 articles), mobile phones (17 articles), phone (121 articles), screen time (164 articles), smartphones (60 articles), social media (309 articles), television (105 articles), and video games (88 articles). The complementary search in ProQuest yielded 5 additional records of doctoral dissertations, thus creating an initial pool of 4682 studies.

### Inclusion and Exclusion Criteria

In the first filtering step, we read the titles and abstracts of the 4682 articles and determined whether they (1) presented an empirical study, (2) were written in English, (3) were published in a peer-reviewed journal (or were a thesis or dissertation), and (4) specifically examined screen time and ASD (as multiple studies were conducted among ASD populations but addressed other negative outcomes, such as sleep problems). In the second filtering step, we read the remaining articles thoroughly and excluded those that did not meet the aforementioned inclusion criteria. In the second step, we also excluded studies that (1) did not report any statistics or reported a single case study, (2) presented a literature review and did not include an empirical study, and (3) had no comparison group, provided that they had a group of participants with ASD (studies without a comparison group were only included if they measured ASD symptoms). Research quality was assessed using the Grading of Recommendations, Assessment, Development, and Evaluations (GRADE) approach.^20^

### Data Extraction and Synthesis

Upon review of the final set, we collected all effect sizes (eg, Cohen’s *d*, Pearson *r*, odds ratio [OR], and log OR values) that represented the associations between screen time and ASD. In cases in which no effect size was reported, we calculated the effect size manually using the reported data or available statistics. We calculated Cohen’s *d* using means and SDs or *t* or *F* scores. We calculated log ORs using either reported frequency tables, reported χ^2^ values, or β coefficients of logistic regression models. In cases in which a linear (not logistic) regression analysis was conducted, we transformed the standardized β coefficients to Pearson *r* values using the following conventional formula: *r* = β + 0.5γ, where γ equals 1 when β is positive and 0 when β is negative.^21^

When different effect sizes were reported for different age groups or for different screen types, they were all entered into the meta-analysis. When more than a single effect size was reported within a given age group or screen device type (eg, for different time points or for different assessment tools), we collected the largest effect size available.

Because ASD is typically perceived as a binary variable indicating whether an individual has this diagnosis, we transformed all collected effect sizes into log OR effect sizes. These log OR scores were then used to calculate the meta-analysis and meta-regression of this study. Because nearly half of the collected studies treated ASD as a continuous variable (measuring ASD symptoms), we conducted complementary analyses using continuous effect sizes (ie, Fisher *z* scores instead of log OR scores). These analyses yielded equivalent results (eFigures 5-7 in Supplement 1) that replicated the main results reported in the following meta-analysis and meta-regression results.

Effect sizes were calculated using the R psych package, version 1.9.1 (R Project for Statistical Computing).^22^ Conversion of effect sizes to log ORs was conducted using the R effect size package, version 0.8.6.1 (R Project for Statistical Computing).^23^ The eAppendix in Supplement 1 presents the exact formulas used in this process.

### Statistical Analysis

The univariate meta-analysis was conducted using a random-effects model and the meta-regression analyses were conducted using a mixed-effects model, both via the restricted maximum likelihood estimator for heterogeneity (τ^2^). The meta-regression analysis addressed 3 independent variables, representing the 3 salient features that differentiated the collected studies from one another and thus allowed us to allocate them to distinct clusters. These variables were as follows: (1) screen type (general use of screens, television, video games, computer, smartphones, or social media), (2) age group (children vs adults or heterogenous age groups), and (3) type of ASD measure (clinical diagnosis vs ASD symptoms). The level of statistical significance in all analyses was *P* < .05 (2-tailed), meaning there were no prior assumptions regarding the direction of the results. Publication bias was tested via the Egger *z* test for funnel plot asymmetry.^24^

The age variable comprised a wide range of ages across the various studies, which made the categorization process difficult. However, we observed that approximately half of the studies comprised children aged younger than 12 years, whereas the others included older or heterogenous age groups; we therefore coded age as a binary variable (children vs adults or heterogenous age groups). All analyses were conducted using the R metafor package, version 2.1-0 (R Project for Statistical Computing).^25^ Data analysis was performed in June 2023.

## Results

### Studies Selected

**Figure 1.**
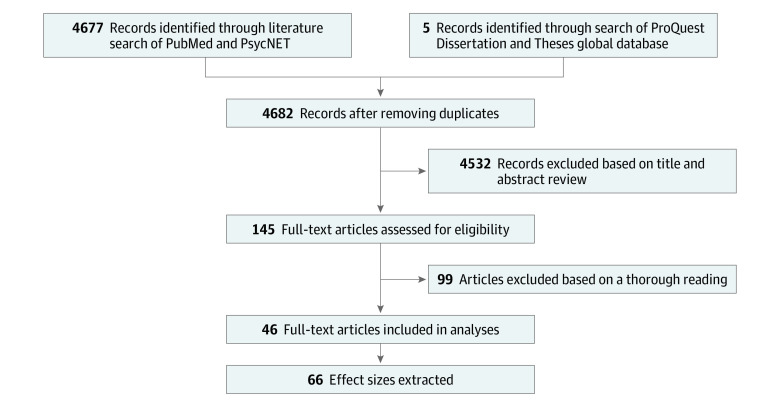
Study Flow Diagram

The initial systematic literature search yielded 4682 records (Figure 1). The first filtering step resulted in 145 studies, and the second filtering step resulted in a final collection of 46 studies^9,10,11,12,13,26,27,28,29,30,31,32,33,34,35,36,37,38,39,40,41,42,43,44,45,46,47,48,49,50,51,52,53,54,55,56,57,58,59,60,61,62,63,64,65,66^ that examined the association between screen time and ASD (Figure 1 and Table 1).

**Table 1.** Characteristics of the Included Studies

Reference	Research design	Screen type	Age range, y	ASD measure	No. of participants	
					Control group	Diagnosed ASD group
Aishworiya et al,^10^ 2022	Longitudinal	General	1-3	Clinical diagnosis	0	229
Alrahili et al,^26^ 2021	Cross-sectional	General	4-6	ASD symptoms	0	308
Berard et al,^27^ 2022	Cross-sectional	General	12-17	ASD symptoms	0	64
Cardy et al,^28^ 2021	Cross-sectional	General	1-19	Clinical diagnosis	287	127
Chen et al,^29^ 2021	Cross-sectional	General	3-5	ASD symptoms	28 586	875
Chonchaiya et al,^30^ 2011	Cross-sectional	Television	1-4	Clinical diagnosis	84	54
Dahlgren et al,^31^ 2021	Longitudinal	Television	9	Clinical diagnosis	88	88
Dahlgren et al,^31^ 2021	Longitudinal	Video games	9	Clinical diagnosis	88	88
Davis et al,^32^ 2023	Cross-sectional	Video games	9-12	Clinical diagnosis	75	73
Dong et al,^33^ 2021	Cross-sectional	General	1-4	Clinical diagnosis	57	101
Dong et al,^34^ 2021	Cross-sectional	General	1-7	ASD symptoms	0	193
Engelhardt et al,^35^ 2013	Cross-sectional	Television	8-17	Clinical diagnosis	41	49
Engelhardt et al,^35^ 2013	Cross-sectional	Video games	8-17	Clinical diagnosis	41	49
Eyüboğlu and Eyüboğlu,^36^ 2020	Cross-sectional	General	2-6	Clinical diagnosis	63	57
Dreyer Gillette et al,^37^ 2015	Cross-sectional	General	10-17	Clinical diagnosis	45 000	900
Güneş et al,^38^ 2023	Cross-sectional	General	2-6	Clinical diagnosis	241	211
Halladay,^39^ 2021	Cross-sectional	General	3-5	ASD symptoms	0	77
Healy and Garcia,^40^ 2019	Cross-sectional	Computer	9	Clinical diagnosis	55	55
Healy and Garcia,^40^ 2019	Cross-sectional	Television	9	Clinical diagnosis	55	55
Healy and Garcia,^40^ 2019	Cross-sectional	Video games	9	Clinical diagnosis	55	55
Healy et al,^41^ 2020	Cross-sectional	General	6-17	Clinical diagnosis	1380	1411
Healy et al,^42^ 2017	Cross-sectional	General	13	Clinical diagnosis	74	67
Heffler et al,^12^ 2020	Longitudinal	Television	1	ASD symptoms	2081	150
Hill et al,^9^ 2020	Cross-sectional	General	3	ASD symptoms	86	20
Keskin and Örengül,^43^ 2020	Cross-sectional	Smartphone	1-5	Clinical diagnosis	20	17
Keskin and Örengül,^43^ 2020	Cross-sectional	Television	1-5	Clinical diagnosis	20	17
Kheir et al,^44^ 2012	Cross-sectional	Television	3-17	Clinical diagnosis	52	42
Krishnan et al,^45^ 2021	Longitudinal	General	3-8	Clinical diagnosis	65	65
Kuo et al,^46^ 2014	Cross-sectional	Television	12-19	Clinical diagnosis	12	12
Kuo et al,^46^ 2014	Cross-sectional	Video games	12-19	Clinical diagnosis	12	12
Kushima et al,^11^ 2022	Longitudinal	Television	1	Clinical diagnosis	328	83 237
Lin et al,^47^ 2019	Cross-sectional	Computer	1-6	Clinical diagnosis	87	145
Lin et al,^47^ 2019	Cross-sectional	Smartphone	1-6	Clinical diagnosis	87	145
Lin et al,^48^ 2023	Cross-sectional	Television	3-12	Clinical diagnosis	72 535	182
Lin et al,^47^ 2019	Cross-sectional	Television	1-6	Clinical diagnosis	87	145
Lin et al,^47^ 2019	Cross-sectional	Video games	1-6	Clinical diagnosis	87	145
Lu et al,^49^ 2022	Cross-sectional	Smartphone	1-3	ASD symptoms	0	10 075
MacMullin et al,^50^ 2016	Cross-sectional	Computer	6-21	Clinical diagnosis	172	139
MacMullin et al,^50^ 2016	Cross-sectional	General	6-21	Clinical diagnosis	172	139
MacMullin et al,^50^ 2016	Cross-sectional	Social media	6-21	Clinical diagnosis	172	139
MacMullin et al,^50^ 2016	Cross-sectional	Video games	6-21	Clinical diagnosis	172	139
Mazurek and Wenstrup,^51^ 2013	Cross-sectional	Social media	8-18	Clinical diagnosis	178	197
Mazurek and Wenstrup,^51^ 2013	Cross-sectional	Television	8-18	Clinical diagnosis	178	197
Mazurek and Wenstrup,^51^ 2013	Cross-sectional	Video games	8-18	Clinical diagnosis	178	197
Mazurek and Engelhardt,^52^ 2013	Cross-sectional	Video games	8-18	Clinical diagnosis	41	56
Melchior et al,^53^ 2022	Cross-sectional	General	2	ASD symptoms	12 950	0
Menear and Ernest,^54^ 2020	Cross-sectional	General	1-5	Clinical diagnosis	20 478	209
Menear and Ernest,^54^ 2020	Cross-sectional	General	6-11	Clinical diagnosis	20 781	608
Menear and Ernest,^54^ 2020	Cross-sectional	General	12-17	Clinical diagnosis	28 458	894
Millington et al,^55^ 2022	Cross-sectional	Video games	≥16	ASD symptoms	0	57
Montes,^56^ 2016	Cross-sectional	General	6-17	Clinical diagnosis	64 163	1393
Must et al,^57^ 2014	Cross-sectional	Computer	3-11	Clinical diagnosis	58	53
Must et al,^57^ 2014	Cross-sectional	General	3-11	Clinical diagnosis	58	53
Must et al,^57^ 2014	Cross-sectional	Television	3-11	Clinical diagnosis	58	53
Must et al,^57^ 2014	Cross-sectional	Video games	3-11	Clinical diagnosis	58	53
Paulus et al,^58^ 2020	Cross-sectional	Video games	4-17	Clinical diagnosis	19	36
Qu et al,^13^ 2023	Cross-sectional	General	0-17	Clinical diagnosis	2929	98 037
Reynaud et al,^59^ 2022	Cross-sectional	General	≥19	Clinical diagnosis	1652	207
Sadeghi et al,^60^ 2023	Cross-sectional	General	1-3	ASD symptoms	0	64
Suleman et al,^61^ 2023	Cross-sectional	General	3-5	ASD symptoms	200	0
Tandon et al,^62^ 2019	Cross-sectional	Computer	6-18	Clinical diagnosis	47 532	540
Tandon et al,^62^ 2019	Cross-sectional	Television	6-18	Clinical diagnosis	47 532	540
van Schalkwyk et al,^63^ 2017	Cross-sectional	Social media	12-19	Clinical diagnosis	43	55
Wu et al,^64^ 2017	Cross-sectional	General	3-6	ASD symptoms	8900	0
Yang et al,^65^ 2022	Cross-sectional	Video games	≥18	ASD symptoms	351	0
Yao et al,^66^ 2022	Cross-sectional	General	1-3	Clinical diagnosis	74	84

Abbreviation: ASD, autism spectrum disorder.

### Sample Characteristics

The 46 included studies were published between 2011 and 2023, with a total of 562 131 participants. Thirteen studies reported on data collected since the COVID-19 pandemic (Table 1).^13,26,27,28,33,34,36,39,49,59,60,65,66^ Notably, the research design of all 46 studies was observational: 41 were cross-sectional^9,13,26,27,28,29,30,32,33,34,35,36,37,38,39,40,41,42,43,44,46,47,48,49,50,51,52,53,54,55,56,57,58,59,60,61,62,63,64,65,66^ and 5 were longitudinal.^10,11,12,31,45^ Accordingly, the overall research quality of these studies was determined to be relatively low (according to the GRADE approach).^20^

Altogether, the 46 studies reported on 66 effect sizes relevant to the association between screen time and ASD. There were 3 effect sizes for social media (n = 784), 3 for smartphones (n = 10 344), 5 for computers (n = 48 836), 13 for video games (n = 2137), 14 for television (n = 207 972), and 28 for unspecified screen devices or screen activity (n = 343 047; coded as “general use of screens” in this review). Regarding the age of the researched populations, 37 effect sizes were observed among children aged younger than 12 years (n = 266 474) and 29 effect sizes were observed among adults or heterogenous age groups (n = 346 646).

### Meta-Analysis

**Figure 2.**
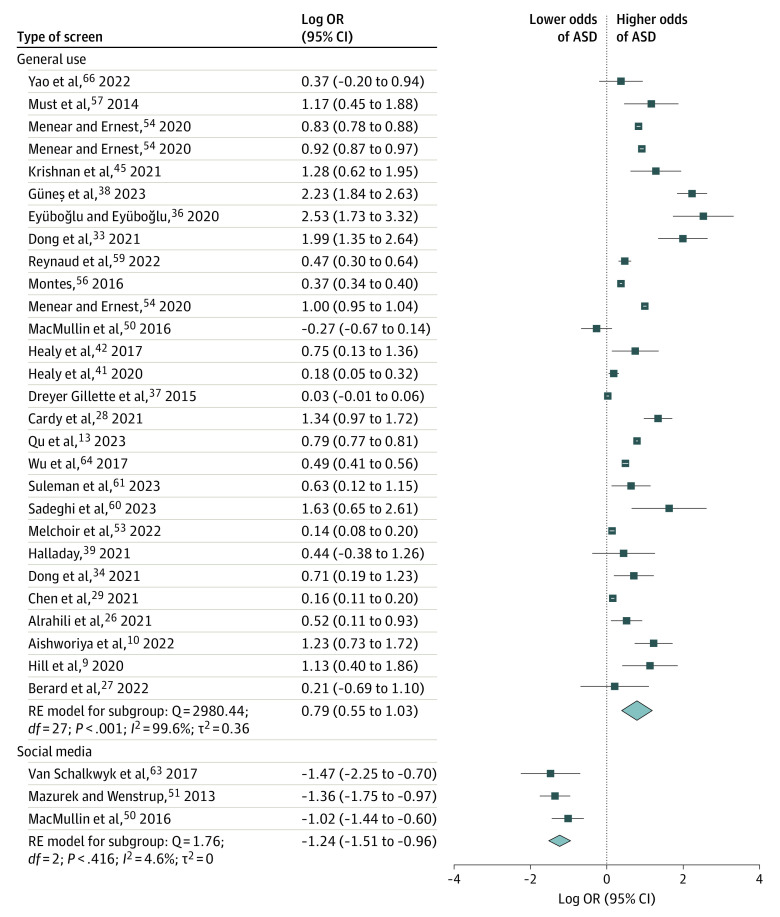
Forest Plot of Effect Sizes for General Use of Screens and Social Media Effect sizes for all screen types are presented in eFigure 1 in Appendix 1.

The meta-analysis of all 66 effect sizes resulted in a significant positive summary effect size (log OR, 0.54 [95% CI, 0.34 to 0.74]; SE = 0.10; *P* < .001; τ*^2^* = 0.58; *Q*_65_ = 6222.68; *P* < .001; *I*^2^ = 99.7%) (Figure 2 and eFigure 1 in Supplement 1). A separate meta-analysis of the 6 longitudinal effect sizes only yielded an equivalent summary effect (log OR, 0.65 [95% CI, 0.26 to 1.05]) (eFigure 4 in Supplement 1) that did not differ significantly from the aforementioned summary effect size (*Q*_m_ = 0.23; *P* = .63).

**Figure 3.**
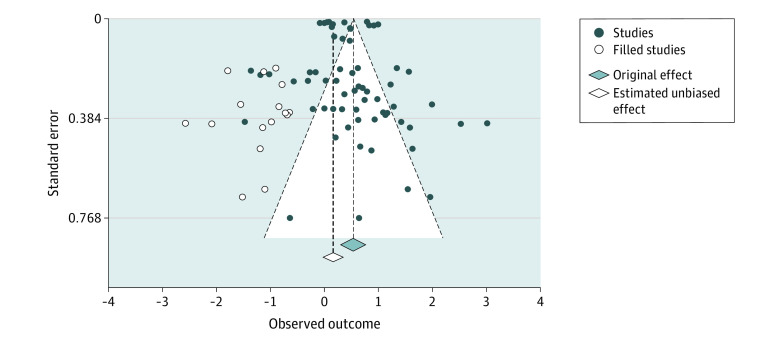
Funnel Plot of the Meta-Analysis Including All 66 Effect Sizes The x-axis represents the observed outcome in log odds ratios. Filled and hollow diamonds represent the results of the meta-analysis before and after trim-and-fill correction, respectively. The diamond centers and corresponding vertical lines represent the value of the summary result. The diamond width represents the 95% CI of the summary result. The white area within the dashed diagonal lines indicates *P* > .05 to *P* > .99; the light gray area outside these lines indicates *P* > 0 to *P* = .05.

An Egger *Z* test for funnel plot asymmetry^24^ suggested a significant publication bias (2.15; *P* = .03). A trim-and-fill correction for this bias^67^ resulted in a substantially decreased and nonsignificant summary effect (log OR, 0.22 [95% CI, −0.004 to 0.44]; *P* = .05) (Figure 3).

### Meta-Regression Analysis

A further meta-regression indicated that all 3 independent variables entered into the model (ie, screen type, age group, and type of ASD measure) contributed significantly to the overall explained variance (*R*^2^ = 0.29; τ^2^ = 0.03; *Q*_e58_ = 2656.55; *P* < .001). However, in terms of β coefficients, only screen type had a significant effect size. That is, the β coefficients for general use of screens (β [SE] = 0.73 [0.34]; *t*_58_ = 2.10; *P* = .03) and social media use (β [SE] = −1.29 [0.51]; *t*_58_ = −2.50; *P* = .01) were significantly different from 0, whereas the effect sizes of television, video games, computers, and smartphones were not statistically significant (Table 2). The association between social media and ASD was negative (log OR, −1.24 [95% CI, −1.51 to −0.96]; *Q*_2_ = 1.76; *P* = .41), whereas the association between general use of screens and ASD was positive (log OR, 0.79 [95% CI, 0.55 to 1.03]; *Q*_27_ = 2980.44; *P* < .001).

**Table 2.** Meta-Regression Analyses of All Effect Sizes and Effect Sizes for General Screen Use

Variable	All effect sizes (n = 66)			General screen use effect sizes (n = 28)		
	β (SE)	*t*_58_	*P* value	β (SE)	*t*_25_	*P* value
Intercept	−0.29 (0.40)	−0.74	.46	−0.13 (0.28)	−0.48	.63
Screen type						
General	0.73 (0.34)	2.10	.03	NA	NA	NA
Television	0.42 (0.37)	1.13	.26	NA	NA	NA
Video games	0.37 (0.37)	0.99	.32	NA	NA	NA
Smartphone	0.44 (0.53)	0.83	.40	NA	NA	NA
Social media	−1.29 (0.51)	−2.50	.01	NA	NA	NA
Age						
Children	0.27 (0.19)	1.42	.16	0.79 (0.25)	3.23	.007
ASD						
Clinical diagnosis	0.30 (0.22)	1.33	.18	0.67 (0.25)	2.69	.001

Abbreviations: ASD, autism spectrum disorder; NA, not applicable.

The most prominent cluster in our review comprised studies addressing general screen use (*k* = 28), which also had the largest heterogeneity (*Q*_27_ = 2980.44; *P* < .001; *I*^2^ = 99.67%). To further examine the positive association evidenced only in this cluster, we conducted a second meta-regression analysis that targeted only the 28 effect sizes reported in the general screen use cluster. Results from the mixed-effects model suggested that both remaining moderators contributed significantly to model variance for age group (β [SE] = 0.67 [0.25]; *t*_25_ = 2.69; *P* = .001) and ASD measure (β [SE] = 0.79 [0.25]; *t*_25_ = 3.23; *P* = .007) (Table 2). Specifically, the effect size for children (log OR, 0.98 [95% CI, 0.66 to 1.29]) was significantly larger than that for adults or heterogenous age groups (log OR, 0.49 [95% CI, 0.19 to 0.79]; *Q*_m_ = 3.94; *P* = .047) (eFigure 2 in Supplement 1). The effect size for the group with an ASD clinical diagnosis (log OR, 0.9 [95% CI, 0.55 to 1.25]) was slightly larger than that for the group with ASD symptoms (log OR, 0.57 [95% CI, 0.32 to 0.82]), but this difference was not significant (*Q*_m_ = 1.14; *P* = .29) (eFigure 3 in Supplement 1).

## Discussion

The goal of this study was to provide an updated systematic review and, to our knowledge, the first meta-analysis of the literature accumulated on the association between screen time and ASD. This review yielded 46 observational studies (5 longitudinal and 41 cross-sectional) with 66 relevant effect sizes. The first meta-analysis of these effect sizes resulted in a statistically significant, although small, summary effect size suggesting that screen time is indeed associated with ASD. This association seemed to be most dominant within studies addressing general screen use among children aged younger than 12 years.

The primary findings of this study may serve as a preliminary warning that supports existing medical recommendations to limit screen use among young children.^15^ This preliminary conclusion corresponds with the few longitudinal studies conducted on this topic to date.^11,12^ According to these studies and the displacement hypothesis described earlier, infancy and early childhood are highly sensitive developmental stages. Therefore, adult caregivers are advised to monitor their children’s screen time and ensure that it does not come at the expense of positive, real-life experiences and relationships, which are essential for the development of communication and emotional skills.

Nevertheless, our primary findings restrict this preliminary conclusion. First, the observational nature of the available studies limits our ability to determine the direction of the association between screen time and ASD. Second, the literature seems to be characterized by a substantial publication bias that challenges the reliability of the observed summary effect size. In fact, when this bias was considered in the analysis, the summary effect size became negligible and insignificant. These findings suggest that the potential negative outcomes associated with screen use may be less severe than commonly believed,^68,69^ especially when they are balanced against other factors such as the specific type of screen use.

As our meta-regression results suggest, the observed effect size for screens disappeared in the separate analyses of studies dedicated to the specific effect sizes of television, video games, computers, and smartphones. Moreover, the association between social media and ASD was negative, thus suggesting that some types of screen use may either protect against ASD or be avoided by users with ASD (depending on the direction of the association). This distinction between various types of screen devices and activities is important because it may offer an explanation for the mixed findings in the existing literature,^17^ and it might facilitate the development of more nuanced guidelines for parents.^68^

Screen use may have both negative and positive outcomes, as described in previous studies.^70,71^ For example, social media use may have some benefits for children with ASD or ASD symptoms, owing to the engagement in interpersonal associations that typically occurs on these media.^14^ This observation replicates, to a certain extent, findings from a related meta-analysis that targeted the association between screen time and language skills.^7^ Although so-called background television had negative effect sizes in this meta-analysis, educational programs and co-viewing had positive effect sizes.^7^ Correspondingly, some video games may be positively associated with intellectual functioning and school performance^72,73,74^; some were even developed specifically for children with ASD to provide emotional support and joyful experiences.^75^

### Limitations

This research has several limitations. First, the heterogeneity in the methodologies and measurements of the studies introduces inconsistencies into the analysis. Second, the correlational nature of the included studies limits our ability to determine the direction of the association, as mentioned earlier. Third, despite our attempt to control for key moderating variables, multiple other potentially confounding variables, such as socioeconomic factors or parental attitudes, limit our interpretation of the findings. Future, high-quality studies are therefore crucially recommended, preferably using objective screen time measurements, longitudinal and experimental designs (eg, through interventions aimed at reducing screen time and examining its implications on ASD symptoms), and comprehensive control of confounding variables. These studies may help determine whether screen use precedes ASD symptom onset or vice versa, and they may generally contribute to more robust understanding of the complex associations between screen use and ASD.

## Conclusions

The results of this systematic review and meta-analysis, including a notable indication for publication bias as well as small and sometimes nonsignificant effect sizes, and the limitations just described suggest that the issue of screen time and ASD is far from being resolved. In fact, the slight superiority (although not statistically significant) of the clinical diagnosis variable over the ASD symptom variable we observed in the meta-regression brings forth the basic obstacle in this field, which relates to the directionality of the association, as discussed at the start of this work. Alongside the displacement hypothesis focused on the potential negative outcomes associated with screens, a large portion of the literature is dedicated to the opposite direction—that is, to the characteristics that draw children with ASD to engage in screen activities.^16,17,18^ As concluded in a previous literature review on this topic, children with ASD seem to “show increased interest in screen viewing… [which] begins at a very young age.”^18^^(p308)^ It is also reasonable to assume that parents of children with clinically diagnosed ASD adopt a relatively permissive position regarding their children’s screen use. It is possible, then, that the observed (bidirectional) association of the current meta-analysis reflects this tendency of children with diagnosed ASD, at least to a certain extent, thus requiring us to continue searching for other explanations for the increasing global rates of ASD. Excessive screen time may indeed come at the expense of positive real-life activities and close familial relationships that could increase ASD risk. However, further research is needed to support this concern, as the increase in ASD prevalence may be attributable to a range of medical, environmental, and societal factors.^1,3,4,76,77^

## Notes

Supplement 1. **eAppendix.** Formulas Used for Conversion of the Various Effect Sizes Into Log Odds Ratios **eFigure 1.** Forest Plot of All 66 Effect Sizes by Screen Type **eFigure 2.** Forest Plot by Age Groups of the 28 Effect Sizes of General Screen Use **eFigure 3.** Forest Plot by the Type of Autism Spectrum Disorder (ASD) Measure of 28 Effect Sizes of General Screen Use **eFigure 4.** Forest Plot of the 6 Longitudinal Studies **eFigure 5.** Forest Plot by Type of Screen of 66 Effect Sizes Using Fisher *z* Scores **eFigure 6.** Forest Plot by Age Groups of the 28 Effect Sizes of General Screen Use Based on Fisher *z* Scores **eFigure 7.** Forest Plot by Type of Autism Spectrum Disorder (ASD) Measure of the 28 Effect Sizes of General Screen Use Based on Fisher *z* Scores

Supplement 2.
Data Sharing Statement
