# Supplementary material for: Screen Time and Autism Spectrum Disorder: A Systematic Review and Meta-Analysis
Source: JAMA Netw Open. 2023 Dec 8;6(12):e2346775. doi: 10.1001/jamanetworkopen.2023.46775 (PMC10709772; doi:10.1001/jamanetworkopen.2023.46775)
Supplement: Supplement 1. — eAppendix. Formulas Used for Conversion of the Various Effect Sizes Into Log Odds Ratios eFigure 1. Forest Plot of All 66 Effect Sizes by Screen Type eFigure 2. Forest Plot by Age Groups of the 28 Effect Sizes of General Screen Use eFigure 3. Forest Plot by the Type of Autism Spectrum Disorder (ASD) Measure of 28 Effect Sizes of General Screen Use eFigure 4. Forest Plot of the 6 Longitudinal Studies eFigure 5. Forest Plot by Type of Screen of 66 Effect Sizes Using Fisher z Scores eFigure 6. Forest Plot by Age Groups of the 28 Effect Sizes of General Screen Use Based on Fisher z Scores eFigure 7. Forest Plot by Type of Autism Spectrum Disorder (ASD) Measure of the 28 Effect Sizes of General Screen Use Based on Fisher z Scores [file jamanetwopen-e2346775-s001.pdf]

Supplementary Online Content

Ophir Y, Rosenberg H, Tikochinski R, Dalyot S, Lipshits-Braziler Y. Screen time and autism spectrum disorder: a systematic review and meta-analysis. *JAMA Netw Open*. 2023;6(12):e2346775. doi:10.1001/jamanetworkopen.2023.46775

eAppendix. Formulas Used for Conversion of the Various Effect Sizes Into Log Odds Ratios

eFigure 1. Forest Plot of All 66 Effect Sizes by Screen Type

eFigure 2. Forest Plot by Age Groups of the 28 Effect Sizes of General Screen Use

eFigure 3. Forest Plot by the Type of Autism Spectrum Disorder (ASD) Measure of 28 Effect Sizes of General Screen Use

eFigure 4. Forest Plot of the 6 Effect Sizes of Longitudinal Studies

eFigure 5. Forest Plot by Type of Screen of 66 Effect Sizes Using Fisher *z* Scores

eFigure 6. Forest Plot by Age Groups of the 28 Effect Sizes of General Screen Use Based on Fisher *z* Scores

eFigure 7. Forest Plot by Type of Autism Spectrum Disorder (ASD) Measure of the 28 Effect Sizes of General Screen Use Based on Fisher *z* Scores

This supplementary material has been provided by the authors to give readers additional information about their work.

© 2023 Ophir Y et al. *JAMA Network Open.*

eAppendix. Formulas Used for Conversion of the Various Effect Sizes Into Log Odds Ratios

From Cohen's *d*:

1. $\log Odds\ Ratio = d\frac{\pi}{\sqrt{3}}$

2. $V_{logOddaRatio} = V_d\frac{\pi^3}{3}$

From Pearson's *r*:

Pearson's *r* is first transformed to *d* and V_d as follows:

3. $d = \frac{2r}{\sqrt{1-r^2}}$

4. $V_d = \frac{4V_r}{(1-r^2)^3}$,

Next, *d* and V_d are converted to log OR and $V_{logOddaRatio}$ using formulas (1) and (2).

© 2023 Ophir Y et al. *JAMA Network Open.*

eFigure 1. Forest Plot of All 66 Effect Sizes by Screen Type

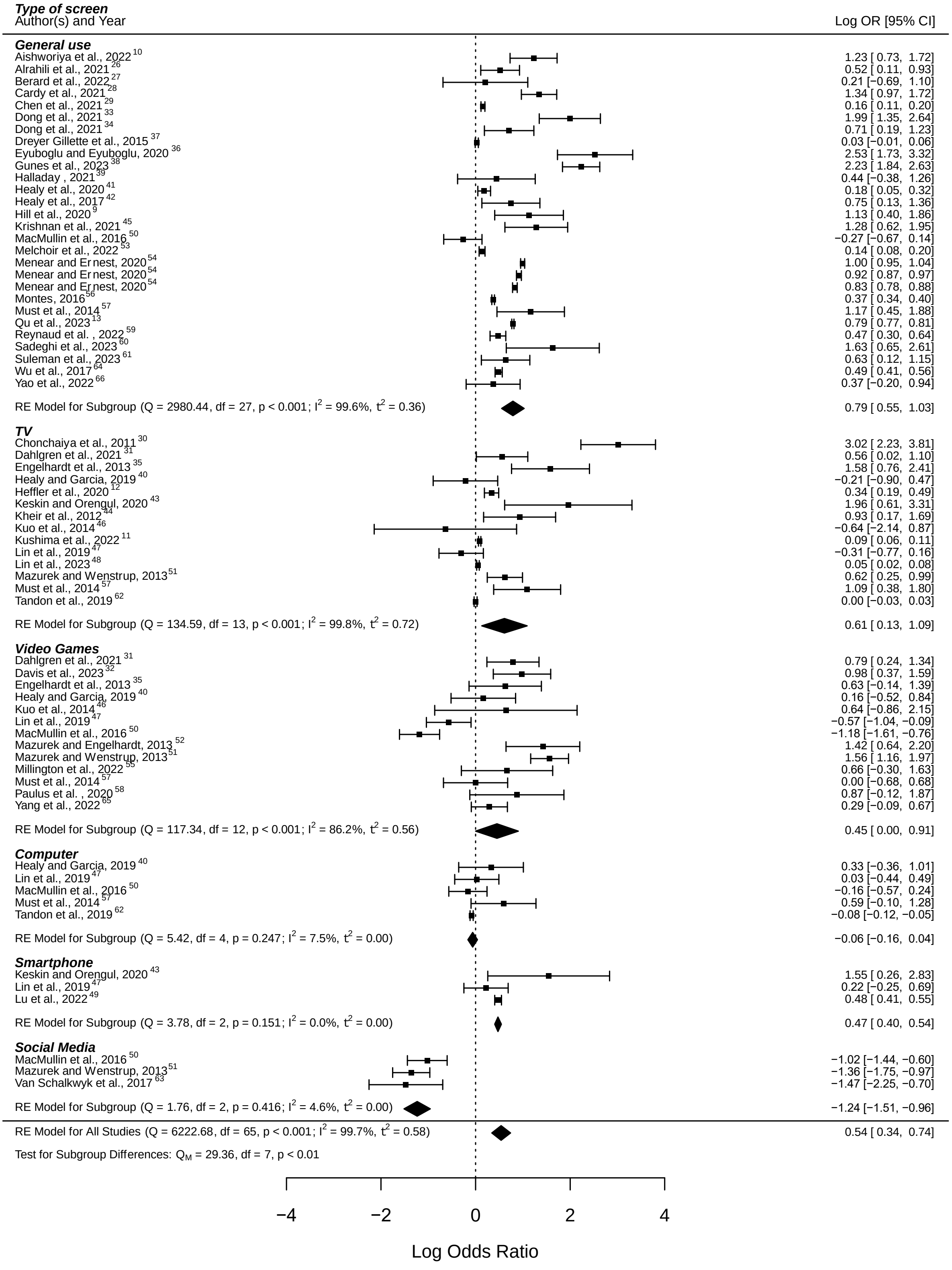

© 2023 Ophir Y et al. *JAMA Network Open.*

eFigure 2. Forest Plot by Age Groups of the 28 Effect Sizes of General Screen Use

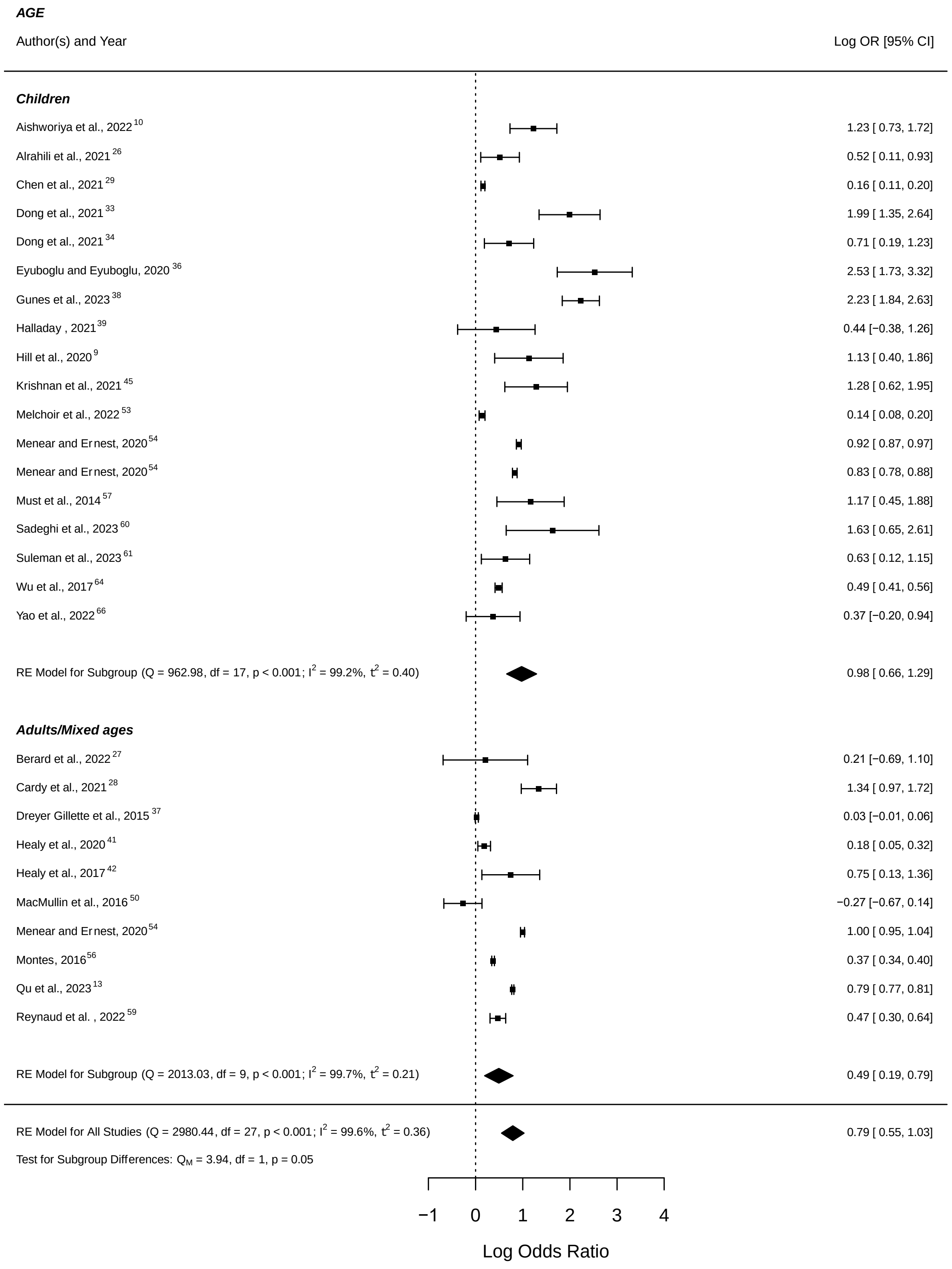

© 2023 Ophir Y et al. *JAMA Network Open.*

eFigure 3. Forest Plot by the Type of Autism Spectrum Disorder (ASD) Measure of 28 Effect Sizes of General Screen Use

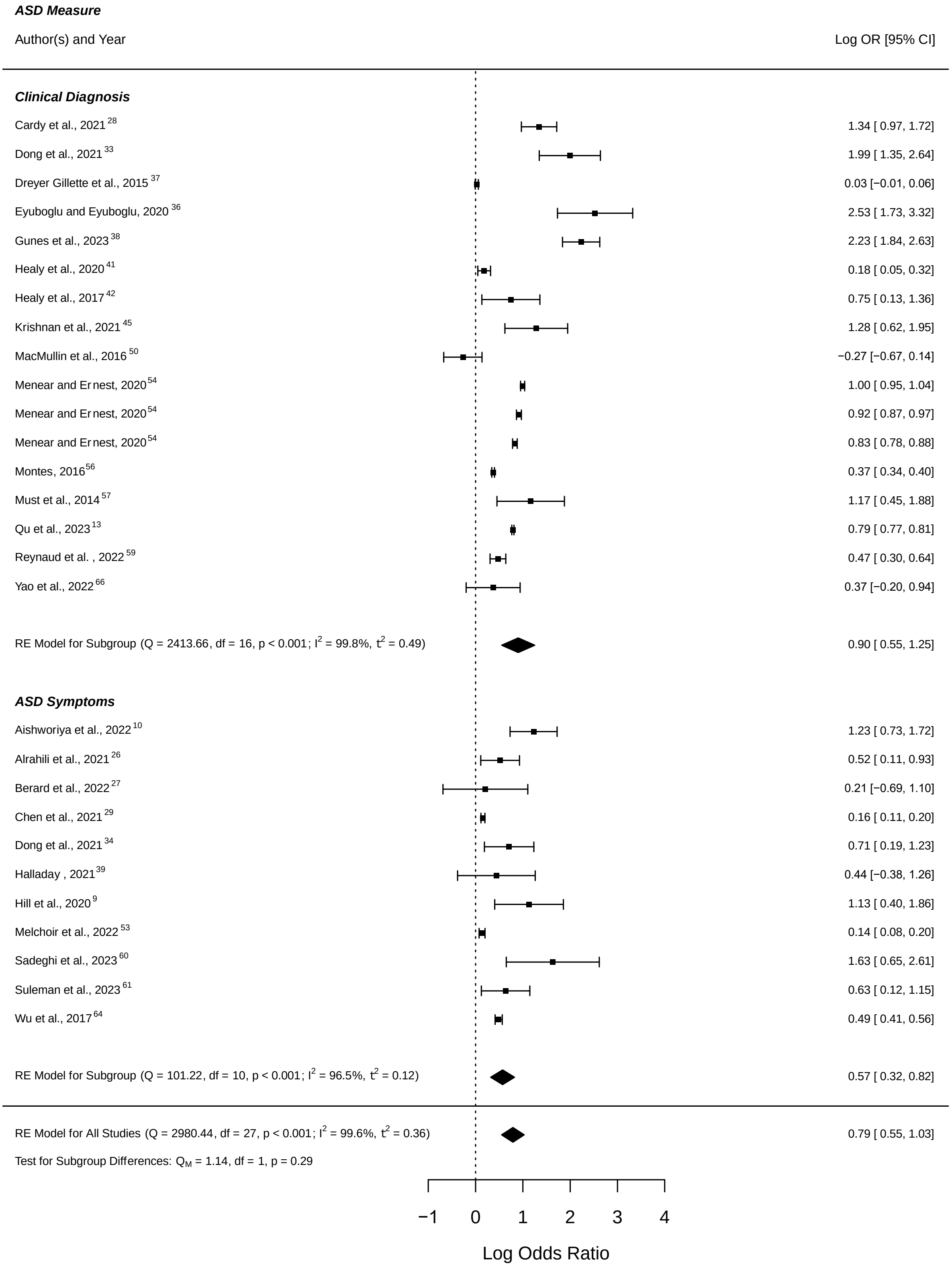

© 2023 Ophir Y et al. *JAMA Network Open.*

eFigure 4. Forest Plot of the 6 Effect Sizes of Longitudinal Studies

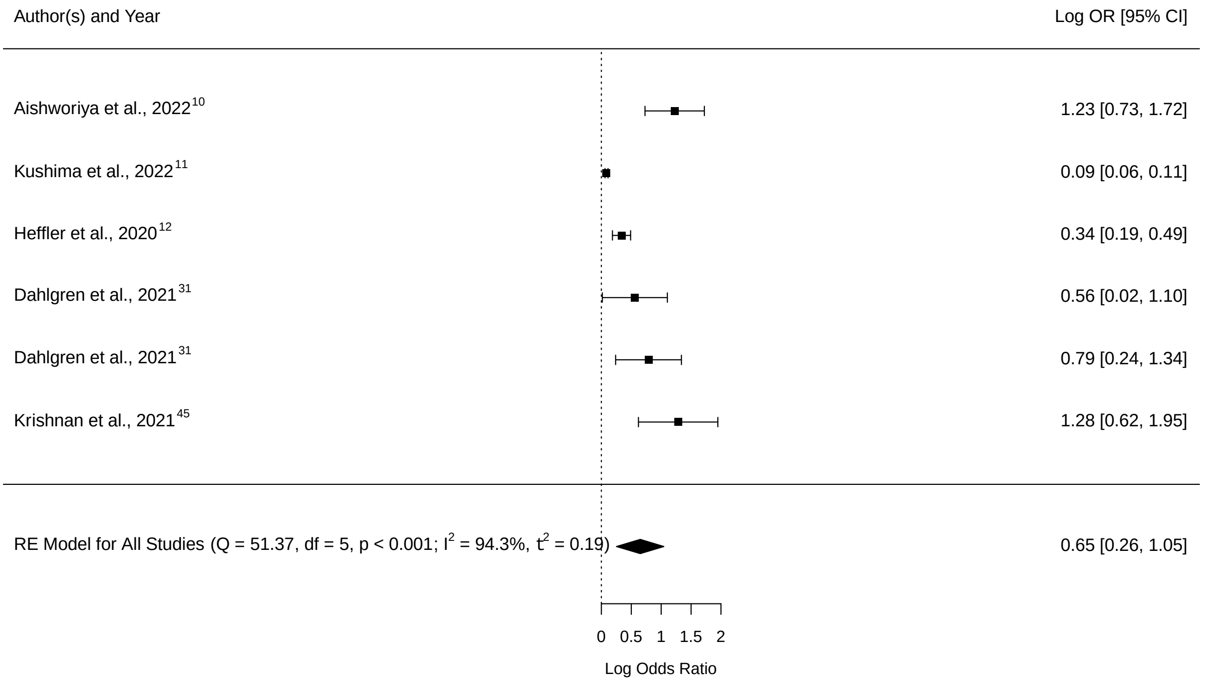

© 2023 Ophir Y et al. *JAMA Network Open.*

eFigure 5. Forest Plot by Type of Screen of 66 Effect Sizes Using Fisher *z* Scores

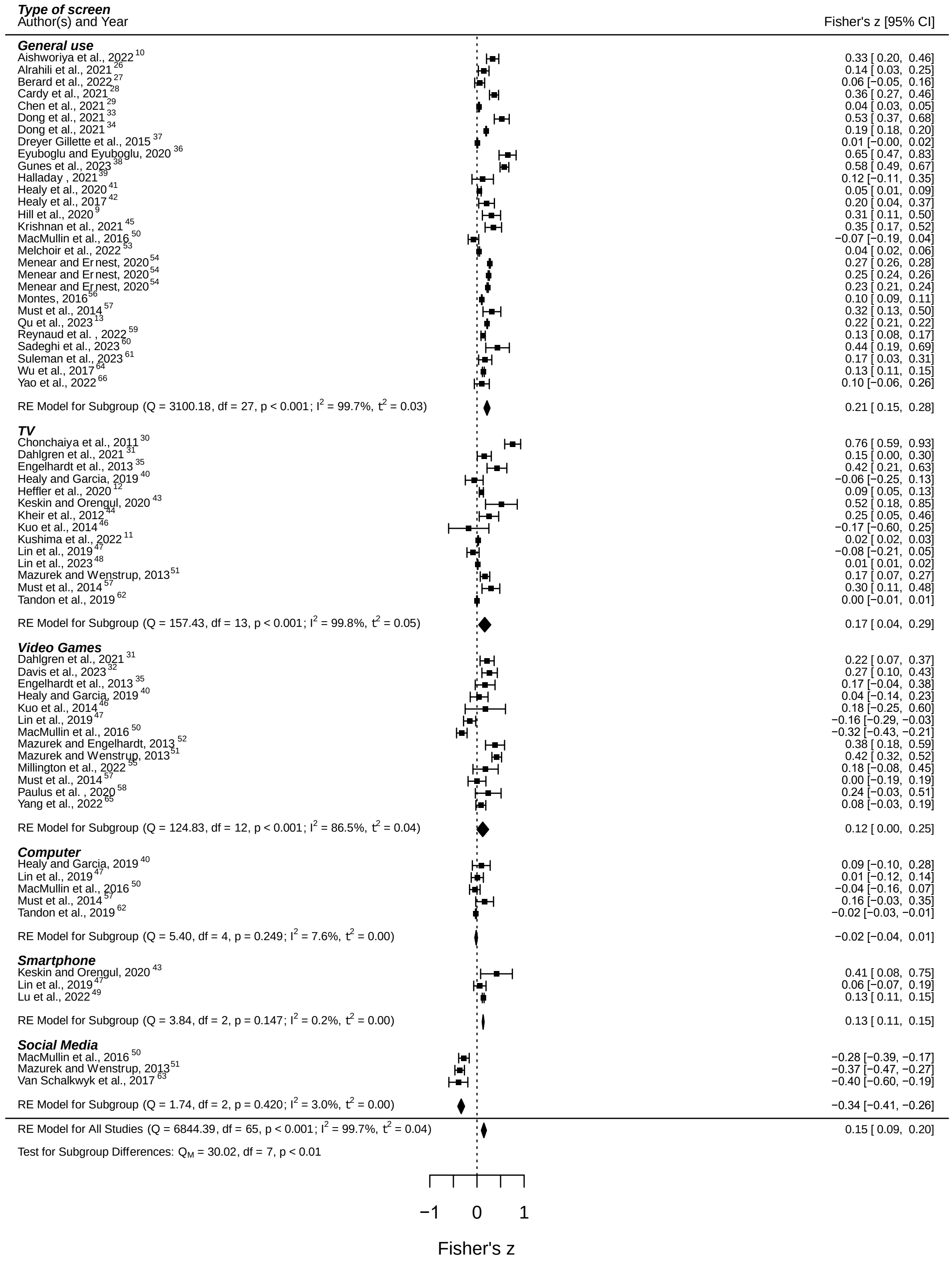

© 2023 Ophir Y et al. *JAMA Network Open.*

eFigure 6. Forest Plot by Age Groups of the 28 Effect Sizes of General Screen Use Based on Fisher *z* Scores

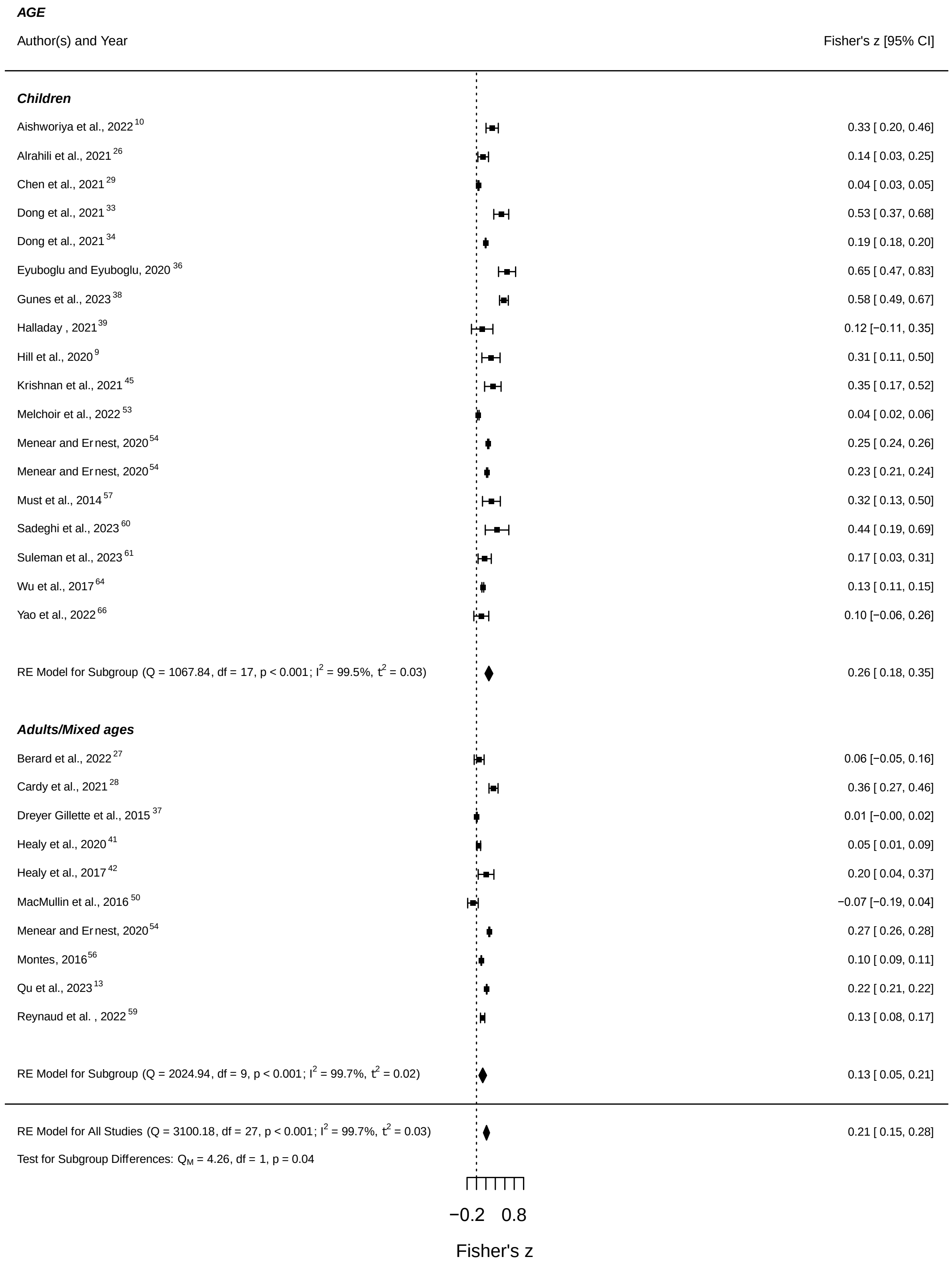

© 2023 Ophir Y et al. *JAMA Network Open.*

eFigure 7. Forest Plot by Type of Autism Spectrum Disorder (ASD) Measure of the 28 Effect Sizes of General Screen Use Based on Fisher *z* Scores

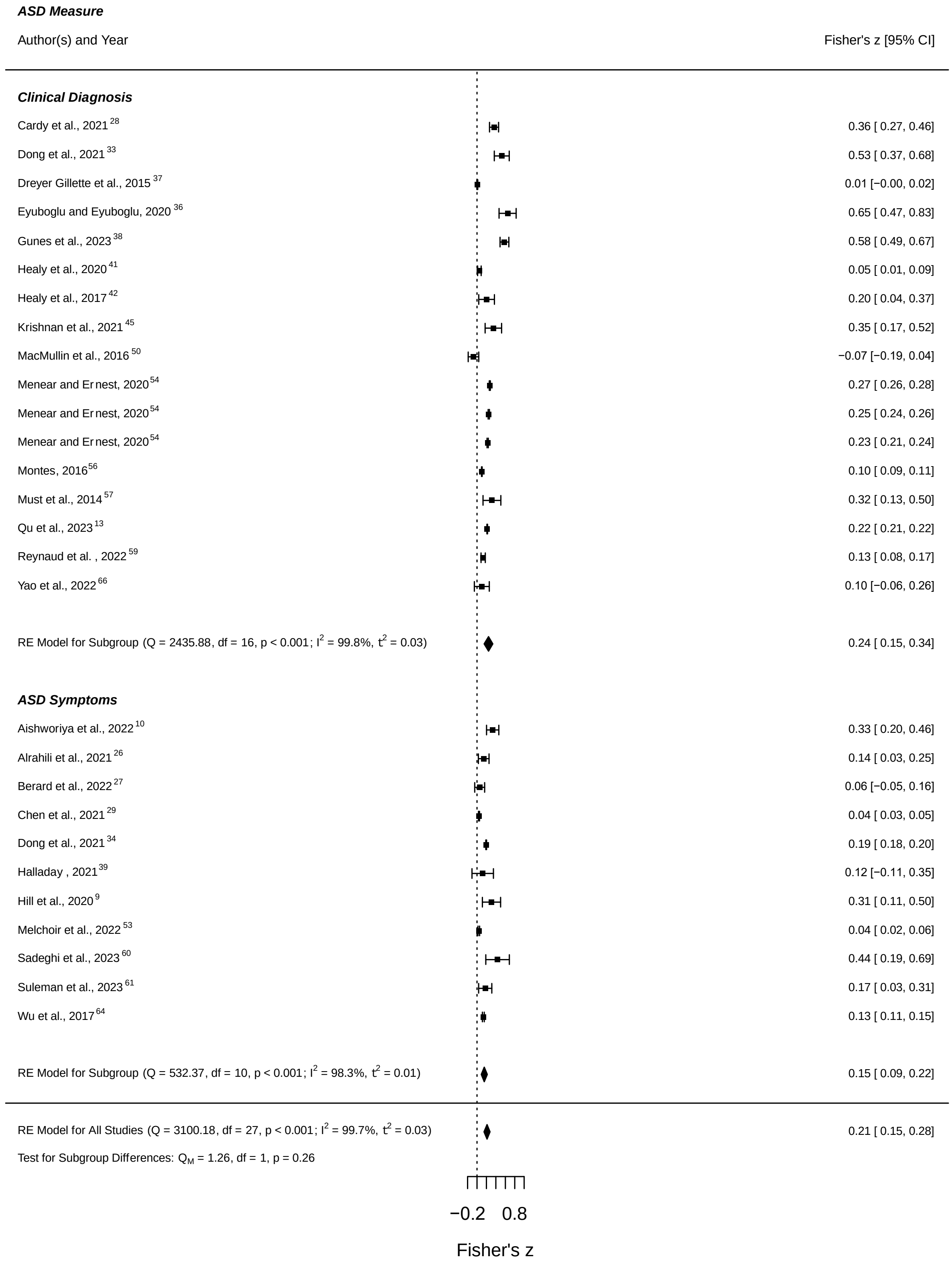

© 2023 Ophir Y et al. *JAMA Network Open.*
