# Supplementary material for: Screen Time and Autism Spectrum Disorder: A Systematic Review and Meta-Analysis
Source: JAMA Netw Open. 2023 Dec 8;6(12):e2346775. doi: 10.1001/jamanetworkopen.2023.46775 (PMC10709772; doi:10.1001/jamanetworkopen.2023.46775)
Supplement: Supplement 2. — Data Sharing Statement [file jamanetwopen-e2346775-s002.pdf]

Data Sharing Statement

Ophir. Screen Time and Autism Spectrum Disorder. *JAMA Netw Open*. Published December 08, 2023. doi:10.1001/jamanetworkopen.2023.46775

Data
Data available: Yes
Data types: Data (not involving human participants)
How to access data: The collection of the relevant statistics that were extracted from these articles to perform the meta-analysis has been uploaded to the Open Science Framework (OSF) and is available at: osf.io/a5tbf.
When available: With publication

Supporting Documents
Document types: None

Additional Information
Who can access the data: to the public
Types of analyses: for any purpose
Mechanisms of data availability: without support
